# The Triple Burden of Malnutrition among Children and Adolescents in Indonesia during the COVID-19 Pandemic: A Narrative Review

**DOI:** 10.1016/j.cdnut.2026.109515

**Published:** 2026-08-12

**Authors:** Marini Amalia Mansur, Vanessa M Oddo

**Affiliations:** 1 Department of Kinesiology and Nutrition, University of Illinois Chicago, Chicago, IL, United States; 2 Department of Nutrition, Hasanuddin University, Makassar, Indonesia

**Keywords:** triple burden of malnutrition, adolescents, children, Indonesia, COVID-19

## Abstract

Children and adolescents in Indonesia are experiencing the triple burden of malnutrition (TBM), resulting from an increasing prevalence of overweight and obesity paired with persistent undernutrition and anemia. The extent to which the coronavirus disease 2019 (COVID-19) further exacerbated TBM within this group, because of lifestyle changes and increased food insecurity, has not been well documented. This narrative review aimed to fill that gap by exploring components of TBM (i.e., undernutrition, anemia, and overweight and obesity), its determinants (e.g., diet), and related nutrition policies and programs among school-aged children and adolescents in Indonesia during the pandemic. We reviewed a total of 10 peer-reviewed articles published between 2021 and 2025 on children and adolescents’ nutrition status and TBM-related determinants. Results suggested that during COVID-19, children and adolescents in Indonesia experienced a high prevalence of undernutrition, overweight and obesity, and anemia. Most children and adolescents also faced higher levels of food insecurity and lower physical activity. The implementation of online learning halted several school-based nutrition programs, including iron‒folic acid supplementation. Nutrition-sensitive programs, such as conditional cash transfers and food assistance, were poorly distributed and contained processed foods, respectively. Nevertheless, some positive changes were also observed, including innovations in the delivery of nutrition education. Dietary changes were mixed. Although some reported a higher consumption of deep-fried foods and snacks, more frequent vegetable consumption and less frequent consumption of sugary drinks were also reported. Future research is needed to understand the mixed findings related to dietary changes and how this contributes to TBM in Indonesia at the national level. Additionally, it is crucial to strengthen children and adolescent nutrition policies and programs that target TBM simultaneously.

## Introduction

More than 390 million children and adolescents worldwide are overweight or obese, with recent trends showing a shift in prevalence from higher- to lower- and middle-income countries (LMICs) [1]. Consequently, LMICs face the triple burden of malnutrition (TBM), or the coexistence of undernutrition, overnutrition, and micronutrient deficiencies within populations [2]. Indonesia, whose population is >280 million and which is the fourth largest worldwide, has experienced an increased prevalence of overweight and obesity [3,4] among children and adolescents, paired with persistent undernutrition and anemia [5,6]. In Indonesia, the prevalence of overweight and obesity among children aged 5 to 12 y was 19.7% in 2023 [7], and among adolescents aged 13 to 15 y was 16.2%, an increase from 11.9% in 2018 [7,8]. The same survey found 11.0% of children and 7.6% of adolescents were underweight; 18.4% of children and 24.1% of adolescents were stunted, whereas 15.5% of adolescents and young adults aged 15 to 24 y were anemic [7]. Thus, the TBM stands to further threaten individuals in a nutritionally vulnerable life stage.

The TBM among children and adolescents is concerning because it is a crucial age for growth and development, as any form of malnutrition in this period could negatively impact health and nutrition in adulthood [9]. For example, systematic reviews show that both undernutrition and overnutrition during childhood and adolescence are associated with later cardiometabolic diseases [10,11]. In addition, obesity may inhibit the reduction of anemia because inflammation caused by obesity can interfere with iron absorption and lead to an individual-level dual burden of malnutrition (overweight and anemia within the same individual), which is prevalent in some developing countries [12,13].

In addition, country-level TBM among school-aged children and adolescents has further strained LMIC economies and policies, as Indonesia, similar to other LMICs, has long focused on interventions to reduce undernutrition [5]. Although some more recent efforts have targeted overweight and obesity [e.g., health promotion programs in the guidelines for Usaha Kesehatan Sekolah (UKS) (school health units)], a holistic approach and nutrition-sensitive interventions remain relatively limited for children and adolescents [5,14].

The COVID-19 pandemic may have further exacerbated the TBM among children and adolescents in Indonesia as a result of lifestyle changes and increased food insecurity [[15], [16], [17]]. However, the extent to which these changes affected TBM within this group in Indonesia is not well documented. This narrative review aimed to fill that gap by exploring the TBM and related nutrition policies and programs among school-aged children and adolescents in Indonesia during the COVID-19 pandemic.

## Methods

Our primary search for this narrative review was conducted using PubMed and Embase to identify peer-reviewed literature. To examine the TBM among children and adolescents, the following terms were used: “nutrition,” “undernutrition,” “overweight,” “obesity,” “anemia,” “food security,” “dietary pattern,” “physical activity,” “children,” “adolescents,” “Indonesia,” “COVID,” “pandemic.” Criteria for inclusion included articles: *1*) published between 2020 and 2026; *2*) participants who were school-aged children or adolescents (6‒19 y); and *3*) included 1 or more of the following outcomes: anthropometric status, anemia, nutrition security, dietary intake/patterns, or physical activity. We excluded the following articles: *1*) review articles and *2*) conference abstracts without full text. Using the aforementioned terms, this search yielded 117 studies from PubMed and 275 from Embase. Covidence was used to identify and remove duplicates prior to screening. After duplicates were removed, a total of 324 studies were screened for relevance based on their titles and abstracts by the first author (MAM). Of those 324 studies, 290 were removed following title and abstract review. A total of 24 articles were further excluded during the first author’s full-text review because they were the wrong population, wrong outcome, or wrong time period. Decisions about full-text inclusion were reviewed by the senior author (VMO). A total of 10 peer-reviewed articles were identified for inclusion (Figure 1). For each included study, the following information was extracted: author, year, location, study design, sample size, age, sex distribution, data collection period (relative to the pandemic), and key findings related to measures of nutritional status (anthropometric indicators, anemia), dietary intake, food insecurity, and physical activity, as applicable.

**FIGURE 1:**
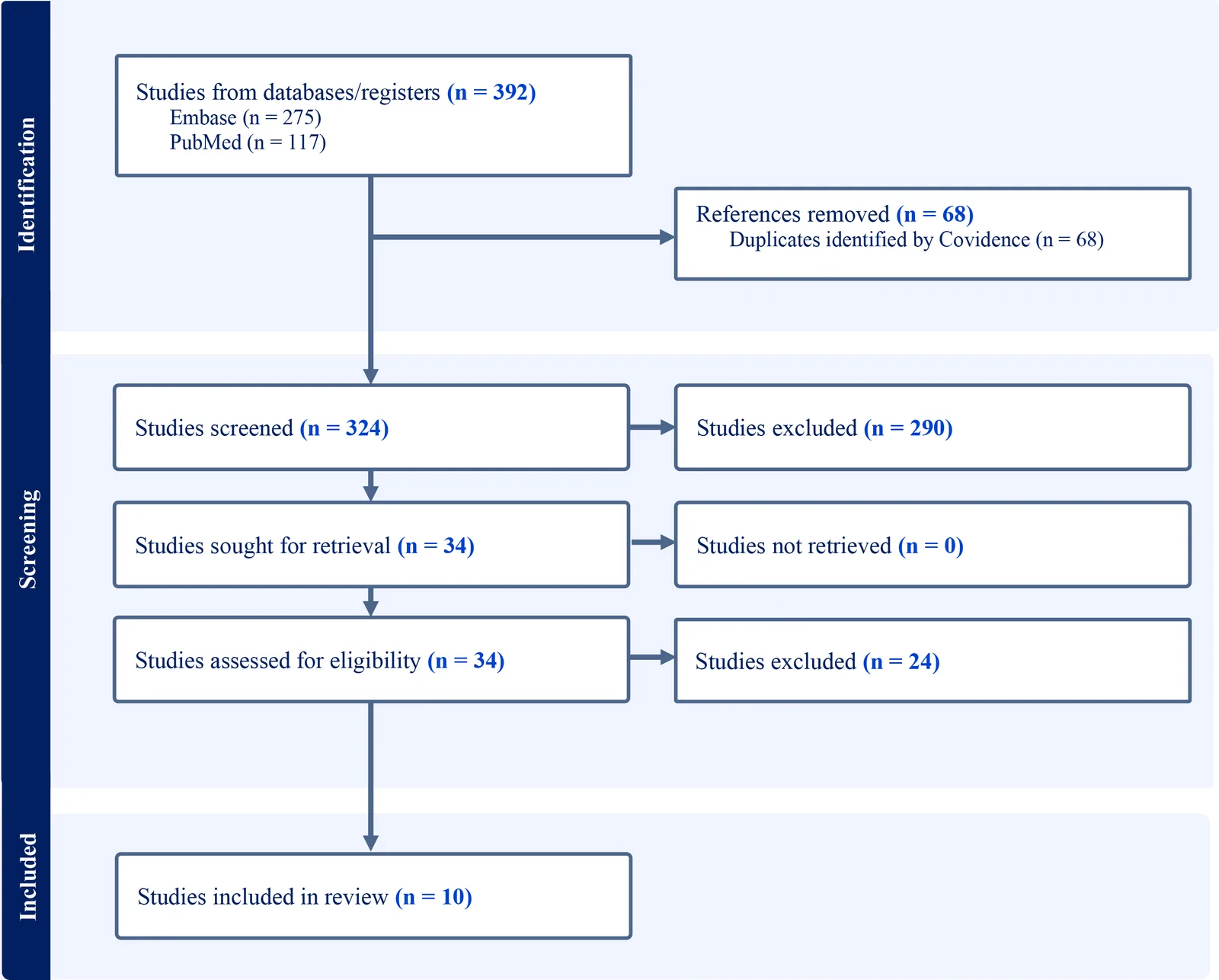
PRISMA chart for the identification and selection of the studies included in a narrative review on the triple burden of malnutrition among children and adolescents in Indonesia during the COVID-19 pandemic.

The following keywords and terms were used to find literature related to nutrition programs and policies for children and adolescents during the pandemic: “policy,” “program,” “adolescent,” “children,” “COVID,” “pandemic,” “nutrition,” “overweight,” “underweight,” “anemia,” “food security,” “dietary pattern,” “physical activity,” “Indonesia.” We found a total of 87 articles in PubMed, 187 in Embase, and 243 in the PAIS (Public Affairs Information Service) index database. The inclusion criteria for articles included the following: *1*) being published between 2020 and 2026 and *2*) containing information on nutrition-sensitive or nutrition-specific programs and policies related to children and adolescents. Of the 517 articles, 47 duplicates were identified, and 470 articles were screened. After the first author screened titles and abstracts, only 1 article discussed nutrition-related policies and programs during the pandemic. Therefore, we expanded our search to the grey literature, which included reports, policy documents, and publicly available government documents in Google and Google Scholar, using the same keywords, which yielded 19 documents in total. Using these search engines, we found 4 journal articles, 9 reports, and 6 government documents explaining available policies and programs, as well as their implementation status. From these documents, we extracted available policies before and during the COVID-19 pandemic (Supplemental Table 1), program name, target population, implementing agency, program components, and implementation status during the COVID-19 pandemic (Supplemental Table 2).

## Results

We included a total of 10 peer-reviewed articles from 9 studies related to TBM in Indonesia during the COVID-19 pandemic (Table 1 [17‒26]).

**TABLE 1:** Characteristics of the studies included in a narrative review on the triple burden of malnutrition among children and adolescents in Indonesia during the COVID-19 pandemic

Author, year	Location	Time	Population (age, sex)	*n*	Study design	Primary outcomes
Asrullah et al., 2024 [17]	Gunung Kidul District, Yogyakarta	Baseline: May to August 2021 Follow-up: May to July 2022	Adolescents (10‒19 y old)	Baseline: 576 Follow-up: 511	Longitudinal	Food insecurity: Household Food Insecurity Access Scale Height and weight: assessed at the respondent’s house using Kenko Stadiometer 250 (height) and GEA EB1622 (weight) Data collection: home visit
Asrullah et al., 2025 [18]	Gunung Kidul District, Yogyakarta	Baseline: May to August 2021 Follow-up: May to July 2022	Adolescents (10‒19 y old)	452 (222 Boys and 230 girls)	Longitudinal	Hemoglobin: measured with HemoCue 201+ Data collection: home visit
Nirmalasari et al., 2023 [19]	Palangkaraya (Central Kalimantan)	September 2021	Adolescents (18‒20 y old)	114 Adolescents (73.7% females)	Cross-sectional	Height and weight: by trained personnel Physical activity: International Physical Activity Questionnaire (IPAQ) Data collection: online using Zoho Forms
Maya et al., 2022 [20]	Pekanbaru (Sumatra)	November to December 2021	Adolescents and early adults (12‒25 y old)	194 Adolescents and young adults (80.5% females)	Cross-sectional	Height and weight: self-reported Data collection: online using Google Forms
Andriyani et al., 2023 [21]	Yogyakarta (urban/suburban areas)	November 2020	Male adolescents (14‒15 y old)	5 Male adolescents	Case-study, mixed-method	Height and weight: measured by researchers using a stadiometer and digital scale Physical activity: accelerometer (ActiGraph wGT3X-BT) during waking hours for 4 d, automated wearable cameras to assess types and contexts of physical activity and sedentary behavior, and a preformatted diary to record activity when the camera/accelerometer was off, as well as bedtime and wake-up time Interview: phone call (mean duration 51 min)
Tan et al., 2021 [22]	Central Java (in a private university)	15‒31 December 2020	University students (18‒21 y old)	107 Students (71.0% females)	Cross-sectional	Height and weight: self-reported; prepandemic weight was recalled by respondents Physical activity: IPAQ-short form Data collection: online using Google Forms
Sekartini et al., 2024 [23]	Indonesia (online data collection)	October to December 2020	Children (6‒12) y old	489 Children (49.7% females)	Cross-sectional	Physical activity: physical activity questionnaire for children Eating habits: questionnaire from SEANUTS Data collection: online questionnaire, recalled by parents of participants
Indriasari et al., 2023 [24]	Indonesia (online data collection, 53% from Sulawesi)	October 2020	Adolescents (10‒21 y old). 72.5% aged >18 y old	772 Adolescents (82.4% females)	Cross-sectional	Height, weight, eating behavior: self-reported Data collection: online using Google Forms
Sarintohe et al., 2024 [25]	Bandung, Manado, Jakarta, Surabaya (5 private schools)	Baseline: October to December 2019 Follow-up: June to September 2021	Adolescents (mean age: 12.02, SD = 0.45)	336 Adolescents (53.7% males)	Longitudinal	Height and weight measurement: Baseline: by school nurses and trained research assistants Follow-up: 129 measured by school nurses, 164 provided by parents, 70 self-reported
Geurts et al., 2024 [26]	15 regencies in Java and Sumatra	September to December 2020	Children (0.5‒12.9 y old) Urban: 48.0% male; rural: 51.3% male	1498 (754 in rural and 744 in urban) Child food habit (CFH) questionnaire (n = 954)	Repeated Cross-sectional (data from SEANUTS II).	Dietary intake: CFH, self-reported COVID-19 questionnaire Food insecurity questionnaire Physical activity questionnaire Data collection: telephone interview, self-reported by parents/guardian

Abbreviations: SEANUTS, Southeast Asian Nutrition Survey.

Studies were conducted in various areas in Indonesia, including Gunung Kidul District (Yogyakarta) [17,18], Palangkaraya (Central Kalimantan) [19], Pekanbaru (Sumatra) [20], Yogyakarta Province [21], and Central Java [22]. Two studies were conducted nationwide using an online questionnaire [23,24], and 2 studies were conducted in multiple regencies in Indonesia [25,26]. Data were collected before the pandemic (October 2019), during the pandemic (October 2020 to September 2021), and after the pandemic (July 2022). The majority of the studies were cross-sectional (*n* = 6) [19,20,[22], [23], [24],^26^], 1 was a case study [21], and 3 were longitudinal [17,18,25]. Most study populations ranged from school-aged children to late adolescence (6‒21 y old); however, 1 study also included children aged <6 y old [26].

Seven articles assessed nutritional status outcomes [BMI-by-age (in kg/m^2^) and anemia] during the COVID-19 pandemic [[17], [18], [19], [20],^22^,^24^,^25^], with 2 articles comparing prevalence during the pandemic with after the pandemic [17,18] and 2 articles comparing before the pandemic with during the pandemic [22,25] (Table 2 [[17], [18], [19], [20],^22^,^24^,^25^]).

**TABLE 2:** Nutritional status among children and adolescents in Indonesia during the COVID-19 pandemic

Authors	Overweight-obese	Underweight	Anemia
Asrullah et al., 2024 [17]	Baseline (During COVID-19) vs. follow-up: Overweight: 12.8% vs. 11.9% ↓ Obese: 7.9% vs. 7.4%↓	Baseline vs. follow-up: 11.0% vs. 8.8% ↓	Baseline vs. follow-up: 23.4% vs. 32.1% ↑^1^
Asrullah et al., 2025 [18]			Baseline vs. follow-up: 21.0% vs. 29.0% ↑ Girls: 31.0% vs. 42.0% Boys: 9.0% vs. 15.0%
Nirmalasari et al., 2023 [19]	Overweight: 22.8% Obese: 7.9%	Severely thin: 12.3% Thin: 13.2%	
Maya et al., 2022 [20]	Overweight: 9.8% Obese: 4.2%	Severely thin: 4.6% Thin: 13.4%	
Tan et al., 2021 [22]	Baseline vs. follow-up: Overweight: 11.2% ↔ Obese: 19.6% ↔ BMI before the pandemic (mean 21.67, SD: 3.64) BMI during the pandemic (mean 22.01, SD: 3.76) ↑	Baseline vs. follow-up: 16.8% vs. 14.0% ↓	
Indriasari et al., 2023 [24]	Overweight: 7.1% Obese: 12.6%	28.4%	
Sarintohe et al., 2024 [25]	Mean zBMI ↓^1^ before vs. during COVID-19 (0.52 vs. 0.43; range ‒3.17 to 2.57 vs. ‒2.75 to 2.45)		

Abbreviations: zBMI, BMI-for-age z-score.

↓ decrease; ↑ increase; ↔ no change

1 *P* < 0.005.

### Overnutrition

Five articles [17,19,20,22,24] examined the prevalence of overweight and obesity during the pandemic, and 1 article examined changes in BMI [25] (Table 2). The prevalence of overweight and obesity was concerning in both the Western and Eastern parts of Indonesia, ranging from 14.0% in Pekanbaru [20] to 30.7% in Palangkaraya [19], higher than the national prevalence reported prior to COVID-19 (11.9% in 2018) [8]. Furthermore, a study by Tan et al. [22], among older adolescents in Central Java, showed that 47.6% of students gained weight during the COVID-19 pandemic (38.3% <5 kg and 9.3% >5 kg). This study also found that among older adolescents, the mean BMI was descriptively higher during the pandemic (mean: 22.01, SD: 3.76) compared with the prepandemic period (mean: 21.67, SD: 3.64) [22]. In addition, a longitudinal study by Asrullah et al. [17] showed a higher prevalence of overweight and obesity during than after COVID-19.

At the same time, a longitudinal study by Sarintohe et al. [25] found a significantly lower mean BMI-for-age z-score (zBMI) when comparing before and during COVID-19 (0.52 compared with 0.43, *P* < 0.005). The reduction of zBMI was significantly higher among those with higher (compared with lower) BMI and among females (compared with males) [25]. These trends reported by Sarintohe et al. [25] differ from what was observed in several other countries, which were higher-income countries, where BMI, and overweight/obesity prevalence increased among children and adolescents [27].

### Undernutrition

Consistent with trends prior to the pandemic, the prevalence of underweight varied by geographic location. Undernutrition remains prevalent among Indonesian adolescents, especially among those in the Eastern region. A study by Indriasari et al. [24], whose data are largely from Sulawesi, and a study by Nirmalasari et al. [19], which was conducted in Central Kalimantan, Eastern Indonesia, report that more than one-quarter of adolescents are underweight (28.4% and 25.5%). This is substantially higher than the national prevalence of underweight reported in 2018, which was 8.7% for adolescents aged 13 to 15 y and 8.1% for adolescents aged 16 to 18 y [8]. At the same time, a cross-sectional study by Tan et al. [22], in Central Java (Western Indonesia), showed a lower prevalence of underweight during COVID-19; the prevalence of underweight decreased from 16.8% pre-COVID–19 to 14.0% during the pandemic [22]. However, this result should be interpreted with caution as the anthropometry data were self-reported and retrospectively recalled for the prepandemic measurement; self-reported BMI data among adolescents in developing countries are prone to measurement bias and inaccuracy, as adolescents tend to underreport their weight [28,29]. Additionally, a study in Gunung Kidul, Yogyakarta, showed a nonsignificant lowering in underweight prevalence during the pandemic in 2021 (11.0%) compared with after the height of COVID-19 in 2022 (8.8%; *P* = 0.58). Despite the limitations in research methods, these findings begin to suggest that the pandemic may have exacerbated existing inequalities between the Western and Eastern parts of Indonesia in underweight prevalence, which is due to enhanced access to government social assistance in the Western part of the country.

### Anemia

Two articles [17,18] from 1 cohort study examined anemia prevalence, using different sample sizes. Both found a significantly higher prevalence of anemia after the COVID-19 pandemic (Asrullah et al. [17], 2024: 23.4% in 2021 to 32.1% in 2022 and Asrullah et al. [18], 2025: 21.0% in 2021 to 29.0% in 2022).

The coexisting and relatively high prevalence of overweight/obesity, underweight, and anemia in this review is concerning, especially because the majority of the participants in several studies [19,20,22,24] were female. Both undernutrition and overnutrition track throughout the life course. In particular, long-term undernutrition in adolescent girls can elevate risk of adverse reproductive outcomes, including low birthweight and preterm delivery, as well as perpetuate intergenerational cycles of malnutrition [30]. Both undernutrition and overnutrition during childhood and adolescence are associated with later-life chronic disease [10,11].

### Determinants of the TBM

#### Food insecurity

Only 2 studies explored food insecurity as an outcome [17,26] (Table 3 [17,26]). Geurts et al. [26] found that food security significantly worsened, in both urban and rural areas, during COVID-19. Likewise, Asrullah et al. [17] found that food security was significantly lower during (59.9%) versus after (67.5%) the pandemic (*P* = 0.003).

**TABLE 3:** Food insecurity among children and adolescents in Indonesia during the COVID-19 pandemic

Authors	Key results
Asrullah et al., 2024 [17]	Baseline (during COVID-19) vs. follow-up: Food secure 59.9% vs. 67.5%^1^
Geurts et al., 2024 [26]	Before vs. during COVID-19: Rural Food secure (26.5% vs. 21.2%); Household insecurity (24.7% vs. 23.7%); Individual insecurity (27.2% vs. 31.4%); Child hunger (21.6% vs. 23.6%) Urban Food secure (31.0% vs. 25.7%); Household insecurity (29.4% vs. 30.0%); Individual insecurity (25.8% vs. 26.6%); Child hunger (13.7% vs. 17.7%)

1 *P* < 0.005.

These findings are consistent with the widely documented economic constraints during COVID-19, due to social distancing measures, high rates of unemployment, and loss of income. A pandemic-era survey in Indonesia reported that >50% of households had ≥1 household member unemployed [31]. In a study by Geurts et al. [26], ∼74% of respondents reported a decrease in household income during lockdown in rural areas and 68.8% in urban areas. In addition, the study reported that 42.3% and 33.6% of respondents in rural and urban areas, respectively, decreased spending on food during the lockdown period [26].

### Dietary intake

Five studies reported outcomes related to dietary intake [17,20,23,24,26], with 1 study comparing differences in diet before and during the pandemic [26] and 1 study comparing during and after the pandemic [17] (Table 4 [17,20,23,24,26]). Some positive changes in dietary intake were reported, including improvements in the frequency of vegetable consumption and fewer “unhealthy” foods. Compared with the prepandemic period, Geurts et al. [26] found that the frequency of vegetable consumption was significantly higher during COVID-19, in both urban and rural areas (*P* < 0.001). This study found that during the pandemic, 80.0% and 79.1% of children and adolescents in rural and urban areas, respectively, ate vegetables 2 to 3 times per week. This is aligned with cross-sectional findings from Maya et al. [20] who found that 60.3% of adolescents had frequent vegetable consumption (>1 time per day) during the pandemic and generally aligned with Sekartini et al. [23] who found that adolescents reported a higher consumption of several healthier food items during the pandemic, including vegetables (24.9% higher), fruits (42.9%), meat and seafood (33.3%), and eggs (46.2%).

**TABLE 4:** Dietary patterns among children and adolescents in Indonesia during the COVID-19 pandemic

Authors	Key results
Asrullah et al., 2024 [17]	Baseline (During COVID-19) vs. follow-up: Inadequate dietary diversity↓^2^ (7.5% vs. 1.6%) Daily intake of high-risk foods (salty food, high-fat food, soft drinks) ↓^2^ (73.1% vs. 32.7%)
Maya et al., 2022 [20]	Snack consumption: >1 time per day 90.7% Fruit consumption: >1 time per day 33.0% Vegetable consumption: >1 time per day 60.3%
Sekartini et al., 2024 [23]	Vegetable consumption: ↑ 24.9% Fruit consumption: ↑ 42.9% Meat and seafood: ↑ 33.3% Egg: ↑ 46.2%, Canned food: ↑ 9.4%, Instant food: ↑ 21.2% High-sugar drinks: ↑ 23.7% Snacks: ↑ 65.8%
Indriasari et al., 2023 [24]	Snacking frequency: ↑ 36% Eating portion: do not know 34% Breakfast frequency: ↔ 46%
Geurts et al., 2024 [26]	Before vs. during COVID-19: Change in Intake Frequency Vegetable consumption ↑^2^ Rural (2‒3 times per week 76.8% vs. 80%); urban (2‒3 times per week 75.6% vs. 79.1%) Deep-fried food ↑^1^ Urban (at least once per day 58.8% vs. 65.9%) Sugar-sweetened beverages↓^3^ (less than once a week/never 22.2% vs. 30.1%) Change in Quantity Rural: ↓ Vegetables (22.7%), fruits (24.1%), meat/poultry/seafoods (30.5%), eggs (22.4%), milk (14.5%), other dairy products (8.1%), canned foods (8.4%), convenience foods (20.6%), processed foods (16.6%), sweetened beverages (20.7%), snacks (22.5%). ↑ Rice/cereals (16.2%), portion size of main meals (24.5%). Urban: ↓ Fruits (22.2%), meat/poultry/seafoods (23.7%), eggs (15.9%), other dairy products (7.1%), canned foods (6.7%), convenience foods (18.3%), processed foods (17.5%), sweetened beverages (17.1%), snacks (22.2%). ↑ Portion size of main meals (23.4%)

↓ Decrease; ↑ increase; ↔ no change.

1 *P* < 0.005.

2 *P* < 0.001.

3 *P* = 0.001.

Furthermore, Geurts et al. [26] found that children and adolescents had significantly less frequent consumption of sugar-sweetened beverages in urban areas during the pandemic [26]; 22.2% of adolescents in urban areas drank sugar-sweetened beverages less than once a week/never compared with 30.1% during the pandemic [26]. Geurts et al. [26] also reported a decrease in the consumption of convenience foods and processed foods both in urban and rural areas.

Nevertheless, higher consumption of some unhealthy foods during the COVID-19 pandemic was also reported. Asrullah et al. [17] found the consumption of "unhealthy" foods (soft drinks, high-fat foods, and salty foods) every day was significantly higher during the pandemic (73.1%) versus after (32.7%). Geurts et al. [26] further reported that the consumption of deep-fried food once per day among adolescents in urban areas was significantly higher after (65.9%) than before (58.8%) COVID-19. Moreover, in cross-sectional analyses, Maya et al. [20] found that 90.7% of adolescents consumed snacks more than once per day. Similarly, Indriasari et al. [24] found that more adolescents had a higher snacking frequency during COVID-19 than those who reported lowering their snacking frequency during this time; snacks consumed by adolescents in Indonesia are often unhealthy and include fried foods (e.g., vegetable fritters, banana fritters, and tofu fritters), instant noodles, and candies [32]. A study by Sekartini et al. [23] also reported higher consumption of some unhealthy foods, including canned foods (9.4%) and snacks (65.8%).

These mixed results may reflect differences in socioeconomic status. However, they are generally similar to a prior systematic review, including studies from mostly high-income countries, in which the consumption of vegetables and snacks was higher, and the consumption of sugar-sweetened beverages was lower during the pandemic [33]. In Indonesia, healthy foods are relatively affordable and obtained not only from local markets but also from door-to-door vendors who sell food using mobile carts and online food delivery [16,34,35]. This enables households to purchase food ingredients without leaving their homes, which was particularly relevant during movement restrictions. This may have facilitated consuming healthier foods during COVID-19. Additionally, during the pandemic, children and adolescents spent more time eating at home, which is typically correlated with eating more vegetables. An in-depth interview among adults that was conducted in Yogyakarta showed that Indonesians generally had high awareness of healthy eating and preferred traditional local dishes [36]. Furthermore, Indonesian adults mainly choose unhealthier foods because of the food environment and convenience, such as limited time for cooking [16,36,37]. However, during the pandemic, parents working from home had more time for meal preparation and showed a greater preference for home cooking, as it was more cost effective and minimized the risk of virus exposure [16]. Moreover, staying at home likely restricted children’s and adolescents’ access to sugar-sweetened beverages. Sugar-sweetened beverages are easily accessible at school, as the school canteen commonly sells sugary drinks, as well as ultraprocessed foods [14,38,39].

### Physical activity

Results from 4 studies among children and younger adolescents [21,23,24,26] showed lower physical activity during COVID-19 (Table 5 [19,21‒24,26]). Sekartini et al. [23] found that mean physical activity was significantly lower during the pandemic (mean: 2.47, SD: 0.75) compared with the prepandemic period (mean: 2.72, SD: 0.71). Similarly, Indriasari et al. [24] found 45% of adolescents lowered their physical activity, and Geurts et al. [26] found 32.5% and 40.9% of children had lower physical activity during COVID-19 in rural and urban areas, respectively. One study that used accelerometer data found high sedentary time among respondents (78% of 592 min of accelerometer wear time on weekdays and 79% of 451 min of wear time on weekends) [21]; although the use of accelerometers provided more robust data (compared with self-report), this study had a smaller sample size and only included males.

**TABLE 5:** Physical activity among children and adolescents in Indonesia during the COVID-19 pandemic

Authors	Key results
Nirmalasari et al., 2023 [19]	Low: 36.0%, moderate: 42.1%, high: 21.9%
Andriyani et al., 2023 [21]	Mean sedentary time Weekdays: 456 min (SD: 145), 78% of total accelerometer wear time Weekend: 344 min (SD: 160), 79% of total accelerometer wear time Mean screen time: Weekdays: 176 min (SD: 101) Weekends: 210 min (SD: 165)
Tan et al., 2021 [22]	PA: active 77.6% Sedentary behavior: >8 h/d 36.4%
Sekartini et al., 2024 [23]	Pre vs. during the pandemic Mean PAQ-C score ↓^1^ 2.72 (0.71) vs. 2.47 (0.75) Active ↓^1^ 82.8% vs. 70.8%
Indriasari et al., 2023 [24]	↓ 45%, ↑ 28%, ↔ 27%
Geurts et al., 2024 [26]	Outdoor PA ↓ (rural 32.5%; urban 40.9%) Indoor PA ↑ (rural 31.3%; urban 38.2%) Use of electronic devices ↑ (rural 25.9%; urban 37.8%)

Abbreviations: COVID-19, coronavirus disease 2019; PA, physical activity; PAQ-C, physical activity questionnaire for children; SD, standard deviation.

↓ Decrease; ↑ increase; ↔ no change

1 P < 0.001.

Findings differed among older adolescents (aged 18‒21 y). Nirmalasari et al. [19] found that 42.1% of adolescents were moderately physically active and 21.9% had excess physical activity. Furthermore, Tan et al. [22] found that 77.6% of adolescents were physically active, whereas only 36.4% were sedentary during COVID-19. These results may reflect the fact that older adolescents had more independence to walk outside without adults. However, both studies used self-reported questionnaires.

### Policies and programs related to children and adolescent nutrition during and after the COVID-19 pandemic

The importance of child and adolescent nutrition is emphasized in various regulations as well as the national development plan of Indonesia [40,41]. However, based on the 19 identified documents (Supplemental Tables 1 and 2), there are limited policies and programs targeting child and adolescent nutrition that focus on the TBM [42]. Before the COVID-19 pandemic, programs that targeted adolescents mainly focused on adolescent girls, particularly in relation to their reproductive health, as the national priority is to reduce stunting during the first 1000 d of life [43]. Furthermore, these efforts aimed to improve adolescent girls’ nutritional status, primarily by preventing anemia, as they will become future mothers [43]. Such programs included iron folic acid (IFA) supplementation and health screening through UKS [43]. In addition, several pilot projects targeting adolescents have been conducted, including the Program Gizi Anak Sekolah (ProGAS) (school-aged children nutrition program) and Aksi Bergizi [43,44].

Of note, IFA supplementation has been implemented in Indonesia since 2016; however, it still faces numerous challenges, such as an unclear funding mechanism at both the government- and school-levels and low coverage [42,45]. The distribution of IFA is carried out in coordination between the community health center (*Puskesmas*) and schools. During COVID-19, the distribution of IFA was disrupted because of school closures and relatively few community health centers prepared standard operational procedures for IFA distribution [46]. Although the Ministry of Health published guidelines for nutrition services during COVID-19, it lacked technical guidelines and caused confusion among health care providers [46,47]. Furthermore, the implementation of online learning caused interruptions in the monitoring of IFA consumption [46]. In addition, nutrition programs under the Ministry of Health faced funding cuts because of budget reallocation that focused on COVID-19 management [46,48]. In-person learning gradually returned in 2022, making iron supplementation more accessible for adolescent girls. However, the postpandemic nutritional recovery is complex, as is the multifaceted nature of anemia prevention.

Previous studies have identified the potential use of technology and social media for nutrition education in Indonesia, as these platforms can achieve high engagement, lower costs, and reach a broader audience [49,50]. Furthermore, a systematic review showed that the use of social media for nutrition education could increase health awareness and improve IFA consumption among adolescents [51]. Despite the aforementioned challenges, COVID-19 created an opportunity to better utilize the internet as a medium for nutrition education. During COVID-19, nutrition communication and education were conducted primarily through webinars, videos, and applications, and this communication received positive feedback [46]. For example, a focus group discussion with the Ministry of Health and health workers in cities/districts on anemia had considerable engagement on YouTube, reaching >10,000 people [46]. This is promising in tandem with a pilot project that combined IFA supplementation and nutrition education to successfully improve IFA intake as well as reduce anemia [45].

Although IFA supplementation has been ongoing since 2016, in the past years, there has been no specific reduction target related to overweight and obesity among school-aged children and adolescents, despite the documented increases in prevalence [43]. However, some ongoing programming includes elements that could support overweight and obesity prevention, including early detection of overweight and obesity, referral for counseling for females aged 15‒49 y, and early detection and monitoring of noncommunicable disease (NCD) risk through Pos Pembinaan Terpadu Penyakit Tidak Menular (POSBINDU) (integrated health posts for NCD prevention) [14]. In addition, UKS are encouraged to conduct health education and services, whereas *Posyandu Remaja* (adolescent integrated service posts) is encouraged to conduct health promotion and health check-ups on reproductive and mental health, nutrition, and NCD prevention [14]. In 2021, the government noted plans for interventions specifically targeting overweight and obesity among children and adolescents, particularly through screening in UKS [52]. COVID-19 caused disruptions in health care services, including in community health centers and schools [46,48]. However, there is no report on the extent to which COVID-19 interrupted services in POSBINDU, *Posyandu Remaja*, or UKS.

Additionally, the government of Indonesia has initiated several school-based intervention pilot programs that focus on the development of healthy eating behaviors [53,54]. These programs are primarily intended to address childhood undernutrition; however, their emphasis on promoting healthy eating behaviors could plausibly contribute to the prevention or reduction of overweight and obesity [53,54]. For example, ProGAS was implemented from 2016 to 2019 in disadvantaged and remote districts with funding from the Ministry of Education and Culture, support from the private sector, and technical support from the World Food Programme [53,55]. ProGAS focused on the provision of healthy breakfast, nutrition and personal hygiene education, and behavior change [[53], [54], [55]]. The program improved nutritional knowledge [54], but was terminated in 2020 due to COVID-19 and limited resources [53].

Aksi Bergizi (nutritious action) is a pilot program that was launched in 2018 in Central Java and West Nusa Tenggara Provinces [53,56] among school-aged children and adolescents [31]. This program included several components, such as weekly IFA supplementation for adolescent girls, nutrition education to improve knowledge, attitudes, and self-efficacy on healthy eating and physical activity, and a Social and Behaviour Change Communication Strategy to improve dietary practices and physical activity [56]. In addition, this program included multisectoral district and provincial government personnel training related to adolescent nutrition and health, as well as training for teachers, health workers, and adolescents [44].

School closures in 2020 disrupted Aksi Bergizi activities, especially breakfast, supervised weekly IFA supplementation, nutrition education, and physical exercise at the junior and senior high school levels [57]. School closures also postponed the monitoring and evaluation of the program [57]. Yet, schools developed innovative approaches to continue, such as involving peer supporters to encourage IFA tablet consumption and home visits to distribute IFA tablets [57]. Moreover, radio talk shows, social media competitions, and online quizzes were used to engage adolescents online and provide nutrition education [57].

The Indonesian government took steps during the COVID-19 pandemic to try to mitigate the unintended consequences of school closures and higher food insecurity in Indonesia. For example, they expanded social protection programs such as Program Keluarga Harapan (conditional cash transfer program), Kartu Sembako (food assistance program), Bantuan Pangan Non-Tunai (noncash food assistance) , direct cash assistance, and a national program for community empowerment [14,48,58,59]. However, few families relied on government assistance because of poorly targeted distribution [58,59], and the food distribution program contained a range of ultra-processed foods, including instant noodles, canned products, processed sauces, and vegetable oil [14].

Improving policies related to physical activity [14,42], food marketing and labeling are also warranted. In 2017, based on Presidential Instruction Number 1 in 2017, “GERMAS” (*Gerakan Masyarakat Hidup Sehat*/Healthy Living Community Movement) was launched, which included instructions to increase the activities of UKS and increase physical activity/sports in schools [60]. However, no detailed technical guidelines were developed. To our knowledge, there are also no policies targeting active transportation that could encourage physical activity among adolescents, nor does the built environment (e.g., cycling paths) support such activity [14]. In addition, policies related to food marketing and labeling, subsidization of healthy diets, and taxes on unhealthy foods are absent despite the fact that there is a relation between the food environment and healthy eating [14,42,61]. A qualitative study revealed that during COVID-19, increased screen time and exposure to food advertisements resulted in demand for foods consumed outside the home [16].

In conclusion, the effect of COVID-19 on the TBM among children and adolescents in Indonesia is varied, likely reflecting ongoing geographic and socioeconomic inequalities in the country. Undernutrition and anemia remain prevalent, especially in less-resourced regions and among more socioeconomically disadvantaged populations. Overweight and obesity are also prevalent. There were mixed findings as it relates to key determinants of TBM. During the pandemic, some children and adolescents had healthier dietary patterns and slightly improved BMI indicators. However, unhealthy dietary patterns also persisted, food insecurity worsened, and studies suggested lower physical activity, particularly among children and younger adolescents. Nevertheless, most of the studies included in this review were cross-sectional and relied on self-reported data. Further studies measuring TBM among children and adolescents at the national level are warranted.

Government responses, including the expansion of social protection programs and adaptation of nutrition services, may have mitigated some negative impacts of the pandemic. However, disruptions to IFA distribution may have worsened anemia prevalence, and there remains limited policy focus on children and adolescents as it relates to diet, physical activity, and overweight and obesity prevention. Such policies are critical for preventing increases in the TBM among this vulnerable segment of the population.

## Author contributions

The authors’ responsibilities were as follows – MAM, VMO: conceptualized the review; MAM: conducted all searches, led title/abstract screening, performed the full-text review, and wrote the initial manuscript draft; VMO: reviewed full-text inclusion decisions, edited, and reviewed the full manuscript; both authors read and approved the final manuscript.

## Data availability

Data described in the manuscript are publicly and freely available without restriction.

## Declaration of Generative AI and AI-assisted technologies in the writing process

During the preparation of this work, the first author, MAM, used ChatGPT in order to assist with grammatical mistakes. After using this tool/service, the author(s) reviewed and edited the content as needed and take(s) full responsibility for the content of the publication.

## Funding

The authors reported no funding received for this study.

## Conflict of interest

The authors report no conflicts of interest.
