# Supplementary material for: The Triple Burden of Malnutrition among Children and Adolescents in Indonesia during the COVID-19 Pandemic: A Narrative Review
Source: Curr Dev Nutr. 2026 Aug 12;10(9):109515. doi: 10.1016/j.cdnut.2026.109515 (PMC13631646; doi:10.1016/j.cdnut.2026.109515)
Supplement: Supplementary file 1 [file mmc1.docx]

**Supplementary Table 1**. Available Policies in Indonesia Related to Child and Adolescent Nutrition Before and After the COVID-19 Pandemic

| **Before COVID-19** | | **After COVID-19** | |
| --- | --- | --- | --- |
| **Policy** | **Main Component** | **Policy** | **Main Components** |
| Presidential Regulation No. 42 year 2013 about National Movement to Accelerate Nutrition Improvement | - Focuses on accelerating nutrition improvement during the first 1,000 days of life. - Adolescents are one of the target populations of the National Movement for the Acceleration of Nutrition Improvement. | Decree of The Minister Of National Development Planning / Head of The National Development Planning Agency Number Kep 124/M.PPN/HK/10/2021 Concerning The Establishment of The National Action Plan for Food and Nutrition For 2021–2024 | - Emphasizes the triple burden of malnutrition as main nutrition problem in Indonesia, including increase of overweight and obesity prevalence among school-aged children and adolescents, along with the existence of undernutrition (wasting and stunting) and micronutrient deficiencies. - COVID-19 has caused major challenges in improving nutrition, due to social distancing policies. - One indicator addressing school-aged children and adolescents’ nutritional status, such as the percentage of school-aged children and adolescents (7-18 years old) with obesity who receive screening for non-communicable diseases. |
| President of Republic of Indonesia. Presidential Regulation No. 83 year 2017 about Strategic Policy of Food and Nutrition | - Strategic policy for food and nutrition development that includes food availability, food accessibility, food utilization, community nutrition improvement, and strengthening the food and nutrition institution. - The community nutrition component includes improving adolescent nutritional status. |  |  |
| National Action Plan for Food and Nutrition 2015–2019 | - Emphasizes the importance of adolescent girls’ nutrition in stunting prevention, as well as the role of multisectoral intervention. - Some programs targeting school-aged children and adolescents including iron-folic acid (IFA)/ multi-micronutrient (MMS) supplementation and weight monitoring. |  |  |
| President Instruction No. 1 in 2017 “GERMAS” (*Gerakan Masyarakat Hidup Sehat*/ Community Movement or Healthy Living) | - Reinforces multi-sectoral action in promotive and preventive measures for non-communicable diseases. - Encourages the Ministry of Education and Culture to enhance the implementation of School Health Unit (UKS) activities and to strengthen physical activity and sports programs in schools and other educational institutions, through both curricular and extracurricular activities. |  |  |

**Sources:** (40-43), (52), (60)

**Supplementary Table 2.** Programs Addressing Child and Adolescent Nutrition in Indonesia and the Implementation during the COVID-19 Pandemic

| **Program** | **Target Population** | **Nutrition Problems Addressed** | **Implementing Agencies and Main Components** | **Implementation During COVID-19** |
| --- | --- | --- | --- | --- |
| Iron Folic Acid (IFA) supplementation | Adolescent girls age 12-18 years old | Anemia | - Implemented by Ministry of Health. - Several initiatives were implemented in collaboration with non-governmental organizations (NGOs), including the MITRA Youth program. MITRA Youth was conducted in 20 districts in East Java and East Nusa Tenggara to increase access and consumption of IFA. This program includes advocacy with district health offices and health centres for the provision of IFA supplements. | - Approximately 15% budget cut in the food supplement. - Budget reallocation caused fewer to no services of IFA treatment for adolescents in community health centers. - In a case study report, only three out of eight districts have standard operational procedures (SOP) for IFA distribution during COVID-19 pandemic, while four of eight district postponed IFA distribution. - The Ministry of Health published guidelines for nutrition services during COVID-19, however, there was some confusion among health care providers on technical aspects. - Health and nutrition education was conducted primarily through online platform and received positive feedback. |
| ProGAS (pilot program) | Primary school children in disadvantage and remote districts in five provinces (East Nusa Tenggara, Maluku, Papua, West Papua, and Banten) | Undernutrition (focus on development of healthy behavior) | - Started in 2016 with funding from Ministry of Education and Culture, support from private sectors, and technical support from the World Food Program. - Aimed to promote healthy behavior, improve nutritional intake, and enhanced ability to learn. - Provision of healthy breakfast, nutrition and personal hygiene education, deworming tablets, school gardens, support for local production, and behavior change. | - Discontinued in 2020 due to the COVID-19 pandemic and resource constraints. |
| Aksi Bergizi (pilot program) | School-aged children and adolescents from junior and senior high school in food insecure area in Central Java and West Nusa Tenggara | Iron deficiency and anemia; undernutrition; improvement of dietary intake and physical activity. | - Pilot program begun in 2016, initiated by UNICEF, government of Indonesia, and other partners. - Weekly iron folic acid supplementation (WIFAS) for adolescent girls that is consumed with home-brought breakfast to minimize side effects. - Multi-sectoral nutrition education to improve knowledge, attitudes and self-efficacy on healthy eating and physical activity. - Social and behavior change communication strategy to improve dietary practices and physical activity. Includes advocacy; capacity strengthening for district officials, health workers, teachers, and student peers; school mobilization activities; and social media, writing, and video production training. | - Increased food insecurity during COVID-19 pandemic caused difficulties in bringing breakfast. - School closures in 2020 disrupted Aksi Bergizi activities. - Peer support activities were continued despite school closures. - Peer support helped the distribution of IFA tablet through home visits and encouraged consumption through messaging. - School closures also postponed the monitoring and evaluation of the program. - Radio talk shows, social media competition, and online quizzes were used to engage adolescents online and provide nutrition education. - In 2023, Aksi Bergizi was expanded and added obesity prevention components, such as strengthening the local school and health system. |
| Health Screening | Individuals aged 15 and above and adolescents | Overweight and obesity | - Overweight and obesity screening for women aged 15-49 years old and referral for counselling. - Early detection and monitoring of NCD risk factors through Integrated Posts for NCD prevention (POSBINDU) at community level. Early detection including measurement of BMI, blood pressure, cholesterol and blood glucose level. - UKS should promote health education, health services strengthening, and the development of healthy school environment. - *Posyandu Remaja* are encouraged to conduct health promotion and health check-ups on reproductive and mental health, nutrition, and NCD prevention. | - In a case study report, six out of eight districts reported temporary close of community health post during COVID-19. - In March 2020, a majority of schools switched to online learning. |
| Social assistance | Underprivileged Household | Undernutrition | - Keluarga Harapan (PKH) (conditional cash transfer schemes). - Kartu Sembako (food assistance programme). - Non-Cash Food Assistance (BPNT) (food subsidies). - Direct cash assistance (unconditional cash transfer). - National program for community empowerment. | - *Sembako* program beneficiaries were extended to an additional 4.8 million families. - Food assistance packages contained a range of ultra-processed foods, including instant noodles, canned products, processed sauces, and vegetable oils. - Many families reported poor targeting of government assistance. |

**Sources:** (14), (42), (44-48), (53-59)
